# Drugs and high hospitalization rate: are they related?

**DOI:** 10.1192/j.eurpsy.2023.1094

**Published:** 2023-07-19

**Authors:** I. M. Figueiredo, I. Cargaleiro, M. Nascimento

**Affiliations:** Hospital Centre of Psychiatry of Lisbon, Lisbon, Portugal

## Abstract

**Introduction:**

Substance use continues to be an important problem among mental health patients either as main diagnosis or as comorbidity. Acute care visits, including emergency department visits and hospitalizations, related to substance use disorders (SUD) are increasing and can be opportunities to engage individuals to get proper treatment (Suen LW et al. J Gen Intern Med 2022; 37(10):2420–2428).

Both mental disorders and SUD lead to subsequent chronic physical conditions, premature death, suicide or overdose (Bennett AC. Public Health Rep 2019; 134(1):17-26) that can be accidental or not.

24% to 32% of patients with Substance Induced Psychosis develop later a schizophrenia spectrum disorder or bipolar disorder (Starzer MSK *et al*. Am J Psychiatry 2018;175(4):343–350) leading to a chronic use of medication and, in several instances, to a necessity of psychiatric in-patient treatment with long hospital stays and high readmission rates (Khan S. Health Reports (2017) 28(8)3-8).

**Objectives:**

Our goal was to analyze if substance use is associated with higher psychiatric hospitalization rates.

**Methods:**

An independent-samples t-test was run to determine if there were more hospitalizations among patients with substance use. Afterwards, the Cohen’s D was calculated to measure the effect size and to see the magnitude of the experimental effect.

**Results:**

A sample of 2604 in-patient treatment episodes was used. The sample had 1696 female patients, 908 male patients and 823 patients had substance use. We found that patients with substance use had a statistically significant higher hospitalization rate (6.82±5.27) than the ones without it (5.32±4.84), t(1483)=6.945, p<0.001. Cohen’s effect size value (d=.30) suggested a small practical significance.

**Conclusions:**

Our findings go mainly accordingly the literature; we found a significant effect of drugs on readmission rates (Böckmann V *et al.* Front Psychiatry 2019; 10:828) but we might have thought it would be bigger. That could be explain by undiagnosed substance use (refusal to admit the use, drugs not detected on lab tests, not requesting toxic lab tests) or higher sample percentage of confounding comorbid diagnoses.

However, is undoubtable the several risks that drugs bring to our health and so the authors aim to raise awareness among the medical community to the importance being alert for signs of use or psychiatric symptoms to avoid reaching a no turning point. It’s known that doctors frequently fail to diagnose SUD in hospitalized patients and given the linkage between use of certain substances and particular medical reasons for admission, it would be well-advised to search for SUD in certain admission diagnoses (Weintraub E *et al.* Am J Addict 2001;10(2):167-77). Knowledge of substance use behavior may help reducing relapse rates and to reduce the risk of developing a SUD (Andersson HW *et al*. Nordic Journal of Psychiatry, 75:3, 160-169).

**Disclosure of Interest:**

None Declared

